# Stabilization Method as a Tool for Electronic State Spectroscopy

**DOI:** 10.1021/acs.jpca.5c02982

**Published:** 2025-06-24

**Authors:** Pedro A. S. Randi, Paulo Limão-Vieira, Márcio H. F. Bettega

**Affiliations:** † Departamento de Física, Universidade Federal do Paraná, Caixa Postal 19044, Curitiba, Paraná 81531-980, Brazil; ‡ Atomic and Molecular Collisions Laboratory, CEFITEC, Department of Physics, NOVA School of Science and Technology, Univerisdade NOVA de Lisboa, Caparica 2829-516, Portugal

## Abstract

We propose a new systematic approach to distinguish valence and diffuse electronically excited states of polyatomic molecules. This method applies the stabilization technique of Hazi and Taylor [*Phys. Rev. A* **1970**, 1 (1109)] to neutral excited states, using basis set diffuseness as the stabilization parameter. By monitoring how excited states behave under basis set contraction, one can differentiate valence states from Rydberg and mixed valence-Rydberg states, reducing the arbitrariness of previous strategies. Valence states remain stable, whereas the energies of Rydberg and mixed states vary. This approach is compatible with any electronic structure method. To illustrate its applicability, we characterize the singlet excited states of CCl_4_, HCOOH, and 2-chlorotoluene. This provides a valuable tool for investigating the nature of the electronically excited states.

## Graphical abstract

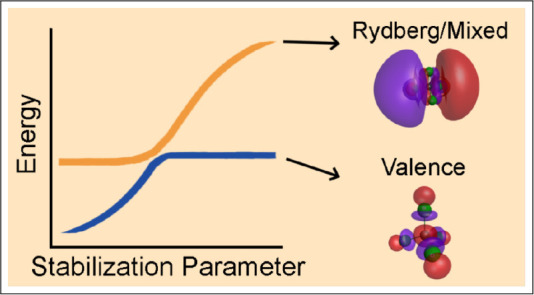

## Introduction

When fundamental particles, such as photons, electrons, and positrons, interact with molecules, they can promote electronic excitation. These electronically excited states play a key role in subsequent physical and chemical reactions across various environments, serving also as a fingerprint for the electronic state spectroscopy of a given molecule. For instance, photon-driven chemical processes are ubiquitous in atmospheric science and astrophysics. Photolysis of molecules present in the atmosphere plays a central role in Earth’s climate^1,2^ while in the interstellar medium similar processes may lead to the formation of prebiotic molecules, which are essential for the origin of life as we know it.^3–5^ In industry, neutral dissociation of molecules through electronic excitation mediated by free electrons^6^ in plasma processing techniques influences the species which are present in the plasma environment.^7^ In the biological media, similar processes may be responsible for the neutral dissociation of important radiosensitizers used in radiotherapy.^8^ Given the broad scientific and technological interest in electronically excited states, numerous electronic structure methods and spectroscopic techniques have been developed to probe their properties over the years (see, for instance, the discussion in ref 9).

Electronically excited states are often multiconfigurational,^9^ meaning that their wave function is described as a linear combination of multiple electronic configurations of the system. Each configuration is associated with one or more promotions of electrons from occupied to unoccupied molecular orbitals. However, low-lying excited states can often be described by one or just a few configurations.^10,11^ Thus, these states are typically characterized based on the nature of the molecular orbitals occupied by the excited electrons in the dominant configurations. States where these orbitals are well localized within the molecule are classified as valence states, whereas those states where the excited electron occupies a diffuse molecular orbital are known as Rydberg states.^12^ Rydberg states are particularly challenging to describe, requiring large and diffuse basis sets due to their extended spatial distribution. In some cases, even low-lying excited states of small molecules have a mixed character, where distinct configurations associated with the excitation of an electron to a valence or Rydberg molecular orbital have similar weights in the description of that given electronically excited state.^11^ Another way to characterize these states is by comparing their electronic spatial radial distribution (SRD) to that of the ground state.^13^ When these values have the same order of magnitude, the state is considered a valence state; otherwise, it may be considered a Rydberg state.

Although the methods for characterizing excited states described in the previous paragraph have been widely used, they remain somewhat arbitrary and subjective. There is yet no complete consensus on what constitutes a well-localized molecular orbital, how much valence configuration must contribute to the description of the electronically excited state for it to be considered a purely valence state, or how closely the SRD of an excited state must match that of the ground state for classification. Orbitals in polyatomic molecules may also exhibit themselves a mixed character, containing both Rydberg and valence components, which further complicates the characterization of the excited states. An additional complication arises when TD-DFT is used to describe the excited state as Kohn–Sham orbitals are not strictly physical molecular orbitals. Consequently, these orbitals can, at best, provide an indication of the character of the electronically excited states. Moreover, single-point vertical excitation energy calculations may not be enough to identify valence states that have neighboring Rydberg states. In these cases, potential energy curves (PECs) may be evaluated to identify the valence states, which is not a direct procedure.^11^ From all of these considerations, we propose a novel approach to systematically distinguish between valence and diffuse states. Our strategy is to apply for the first time the stabilization method (SM) of Hazi and Taylor^14^ to the electronically excited states of neutral polyatomic molecules.

The remainder of this paper is organized as follows: in the next section, the stabilization method and our proposed application are discussed. Then, we apply the proposed method for CCl_4_, HCOOH, and 2-chlorotoluene molecules and discuss the results. Finally, a brief conclusion of this work is presented.

## Stabilization Method

This methodology was originally proposed to characterize resonances in scattering problems.^14^ Resonances are quasibound states of the system embedded in the continuum. When these kinds of problems are treated with a finite basis set, the states obtained are either resonances or states associated with the discretization of the continuum (DC). These different states are distinguished through their stability, regarding the variation of a stabilization parameter. For instance, in the case of electron-molecule resonances, i.e., the capture of the incident electron by the target molecule, the stabilization parameter is often the diffuseness of the basis set employed in bound state calculations of the anion (see e.g., ref 15). While resonances are relatively insensitive to the stabilization procedure, DC states associated with a free electron and a neutral molecule vary considerably as the basis set is compacted or made more diffuse.

Similarly, we apply this rationale to differentiate well-localized valence states from Rydberg and mixed states of neutral molecules. Our proposed approach is to perform a series of electronic structure calculations in which the exponent of the most diffuse Gaussian functions in the basis set is scaled by a stabilization parameter (α). Then, the behavior of the vertical excitation energies obtained as a function of α is thoroughly monitored. This enables for a systematic procedure to evaluate how the diffuseness of the basis set affects each electronically excited state. Well-localized valence states should remain stable, while diffuse Rydberg states should exhibit significant variations. Our approach is based on the understanding that compacted basis sets incorrectly represent Rydberg states, which only converge as the basis set becomes more diffuse.^16^ In contrast, valence states are well localized within the molecule, meaning that the diffuse portion of the basis set has little to no influence on their description. Thus, valence states should be stable as the basis set is compacted. It is important to note that mixed states with a strong Rydberg component are expected to behave like pure Rydberg states. Therefore, the method proposed here is not suitable for distinguishing pure Rydberg states from mixed states with an appreciable Rydberg component.

Here, the singlet states of three test molecules, namely, CCl_4_, HCOOH, and 2-chlorotoluene, are used as test cases of our proposed approach. These systems have been studied previously, and their electronically excited states have been reported along with photoabsorption cross sections.^10,11,17^ CCl_4_ is a suitable first application due to the nature of its low-lying electronically excited states, which are predominantly either valence or Rydberg in character, with minimal mixing.^10^ In contrast, the low-lying electronically excited states of HCOOH have a strong mixing between valence and Rydberg components.^11^ This makes it an interesting system for examining how different mixed states behave within our proposed approach. Finally, 2-chlorotoluene is an example of a larger molecule whose spectrum is denser than the other smaller molecules studied.^17^

To calculate the vertical excitation energies, we performed time-dependent density functional theory (TD-DFT)^18,19^ calculations for the first few singlet states of each molecule. Additionally, for the smaller molecules, calculations with the equation of motion coupled-cluster with singles and doubles (EOM-CCSD)^20–23^ were also performed. These calculations explicitly demonstrate that the procedure that we propose for characterizing valence states is independent of the underlying electronic structure method used. For CCl_4_, calculations were performed using the PBE0 ^24^ functional, while for HCOOH and 2-chlorotoluene, the CAM-B3LYP^25^ and B3LYP^26,27^ functionals were used, respectively. These functionals were chosen for each molecule because they are able to successfully and accurately reproduce the major electronic transitions from the experimental data for the photoabsorption cross sections, as reported in previous studies.^10,11,17^ The aug-cc-pVDZ and aug-cc-pVTZ basis sets^28^ were employed in the calculations, and the exponents of the augmented functions of all atoms were scaled by the factor α. The calculations were carried out at the equilibrium geometry of the ground state, optimized using the default (α = 1) basis set and the same functional as that in the vertical excitation energy calculations. All TD-DFT calculations were performed with the GAMESS computational package^29^ while Psi4 ^30^ was used for the EOM-CCSD calculations.

Before probing the reliability of the methodology, two important points should be noted. First, our proposed approach deliberately relies on the known limitations of compact basis sets in describing the Rydberg states. Therefore, one should ensure that the basis set used in the most compact calculations is indeed compact. In the test cases presented here, this is achieved by scaling the augmented functions of all atoms, rather than selectively scaling only certain atoms (e.g., only C in CCl_4_). Second, in our proposal, the electronically excited state is characterized by the behavior of its vertical excitation energy as the stabilization parameter varies, and no physical information is directly extracted from the plot orbitals themselves. Thus, the complications associated with using Kohn–Sham orbitals in TD-DFT calculations to characterize the excited states are completely avoided in the currently proposed approach.

## Results and Discussion

### CCl_4_

The vertical excitation energy of the electronically excited states of CCl_4_ as a function of the stabilization parameter α, obtained with the TD-DFT/PBE0/aug-cc-pVDZ calculation, is presented in Figure 1. CCl_4_ is a tetrahedral molecule, belonging to the *T*_*d*_ point group. Thus, the electronically excited states may belong to the irreducible representations (IR) *A*_1_, *A*_2_, *E*, *T*_1_, or *T*_2_. The IR *E* is doubly degenerate, whereas *T*_1_ and *T*_2_ are triply degenerate. The vertical excitation energies were calculated in the abelian subgroup of the highest symmetry, *C*_2_ *_v_*. Nonetheless, states are labeled according to the irreducible representation of the *T*_*d*_ point group.

**Figure 1:**
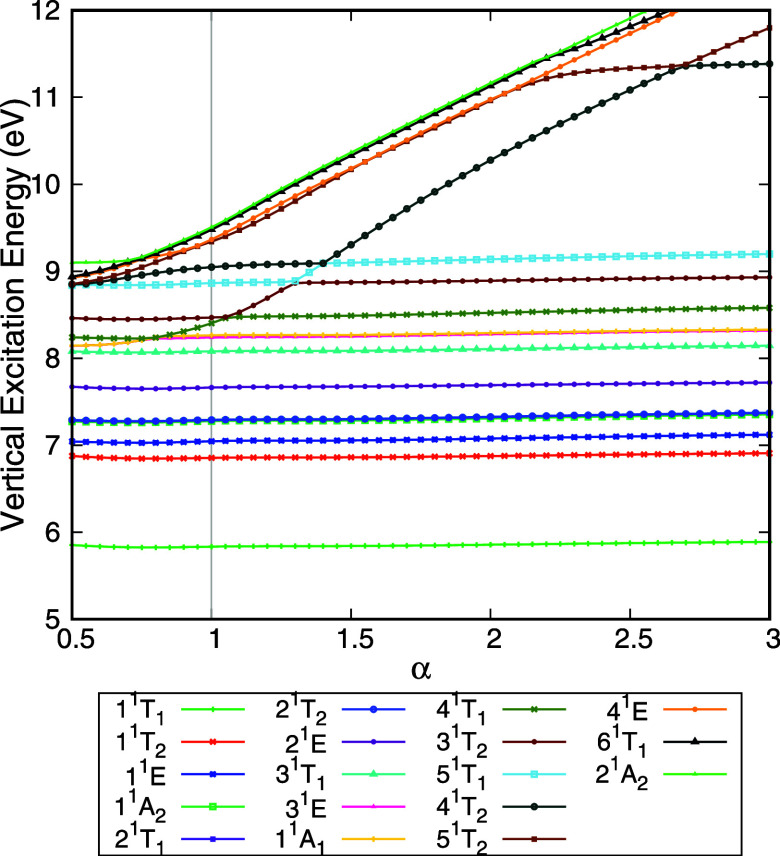
Vertical excitation energy of the singlet electronically excited states of CCl_4_ as a function of the stabilization parameter (α). These results were obtained with TD-DFT/PBE0/aug-cc-pVDZ calculations. The vertical gray line highlights the results obtained with the standard basis set (α = 1) and state labeling is based on the calculations performed with this basis set. States that are stable as the α values vary are considered valence states, while states with a strong Rydberg component vary more dramatically. States 1^1^ *T*_1_, 1^1^ *T*_2_, 1^1^ *E*, 1^1^ *A*_2_, 2^1^ *T*_1_, 2^1^ *T*_2_, 2^1^ *E*, 3^1^ *T*_1_, 3^1^ *E*, 1^1^ *A*_1_, 3^1^ *T*_2_, 5^1^ *T*_1_, and 4^1^ *T*_2_ are valence states, while the remaining states are either Rydberg or mixed states whose Rydberg component is relevant for its description.

From Figure 1, one can observe two behaviors from the excited states as α varies. First, states 1^1^ *T*_1_, 1^1^ *T*_2_, 1^1^ *E*, 1^1^ *A*_2_, 2^1^ *T*_1_, 2^1^ *T*_2_, 2^1^ *E,* and 3^1^ *T*_1_ are stable as α varies. This is the expected behavior of the valence states. States 3^1^ *E* and 1^1^ *A*_1_ exhibit an avoided crossing around α = 0.80 with state 4^1^ *T*_1_, which is due to state 4^1^ *T*_1_ having Rydberg character between α = 0.80 and 1.05. At α = 1.10, another avoided crossing occurs between states 4^1^ *T*_1_ and 3^1^ *T*_2_. As this state is followed from α = 1.10 onward, additional avoided crossings are observed. Moreover, as the basis set is compacted (increasing α), the vertical excitation energy of this state increases. As the basis set is made more diffuse (decreasing α), this state converges to a given value. This behavior is expected for a state in which the Rydberg component plays a significant role in its description, with the vertical excitation energy being sensitive to the diffuseness of the basis set. Thus, state 4^1^ *T*_1_ calculated with the default basis set (α = 1) is not a valence state. Following this reasoning, states 3^1^ *T*_2_, 5^1^ *T*_1_, and 4^1^ *T*_2_ present a valence character, while the remaining states—5^1^ *T*_2_, 4^1^ *E*, 6^1^ *T*_1_, and 2^1^ *A*_2_—have a strong Rydberg component in their description. As a final remark, the small increase in the stabilization plots at α = 0.5 is most likely caused by numerical instabilities in the calculation due to linear dependencies in the most diffuse basis set.

In Figure 2, the vertical excitation energies of CCl_4_, obtained by using the EOM-CCSD method and the aug-cc-pVDZ basis set, are presented as a function of the stabilization parameter α. Due to the high computational cost of EOM-CCSD, the calculations were performed separately for each irreducible representation (IR), according to the*C*_2_ *_v_* point group, and to fewer states. Only the results for the *A*_1_ and *A*_2_ IRs are shown, since with these at least one component of each degenerate state is included in Figure 2. The results are consistent with those obtained using TD-DFT: while valence states are stable as the basis set is compacted, Rydberg and mixed states with strong Rydberg character are highly sensitive. The diffuse states undergo avoided crossings with valence states as α varies, analogously to the case of TD-DFT. This highlights that the procedure proposed here is independent of the choice of the underlying electronic structure method. As is the case for TD-DFT, numerical instabilities affect the calculations. Thus, the results shown in Figure 2 are those for which the calculation is well converged.

**Figure 2:**
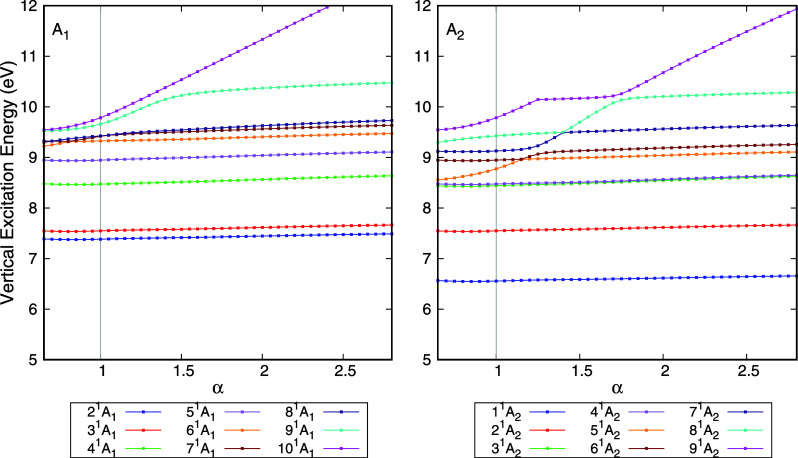
Vertical excitation energy of the singlet electronically excited states of CCl_4_ as a function of the stabilization parameter (α). These results were obtained at the EOM-CCSD/aug-cc-pVDZ level according to the*C*_2_ *_v_* point group. The vertical gray line highlights the results obtained with the standard basis set (α = 1). States that are stable as the α values varies are considered valence states, while states with a strong Rydberg component vary more dramatically.

In Table 1, we present a comparison between the assignment of the excited states following our proposed approach and through an analysis of the diffuseness of the orbital to which the electron is excited in the dominant configurations, as previously described in the introduction of this paper. As one can see, the two approaches agree on the assignment of the valence states. However, as previously mentioned, our method does not distinguish between purely Rydberg states and mixed states. This can be seen in the comparison between the higher lying excited states of the molecule. Nevertheless, the proposed method is able to assign valence states in a systematic way, regardless of the electronic structure method employed and without requiring direct orbital analysis, thereby avoiding arbitrariness and, in the case of TD-DFT, the complications associated with Kohn–Sham orbitals. It is also important to note that since EOM-CCSD is more expensive, fewer excited states were calculated, and the order of these states may change depending on the method and functionals used.

**Table 1:** Vertical Excitation Energy (in eV) and Assignments of the Electronically Excited States of CCl_4_ ^a^

TD-DFT/PBE0/aug-cc-pVDZ				EOM-CCSD/aug-cc-pVDZ				
State	Energy	SM	Orbitals	State	Energy	SM	Orbitals	Exp.^10^
1^1^ *T*_1_	5.834	Valence	Valence	1^1^ *A*_2_	6.556	Valence	Valence	
1^1^ *T*_2_	6.855	Valence	Valence	2^1^ *A*_1_	7.384	Valence	Valence	7.06(1)^b^
1^1^ *E*	7.046	Valence	Valence	3^1^ *A*_1_ + 2^1^ *A*_2_	7.549	Valence	Valence	
1^1^ *A*_2_	7.278	Valence	Valence	3^1^ *A*_2_	8.442	Valence	Valence	
2^1^ *T*_1_	7.289	Valence	Valence	4^1^ *A*_2_	8.475	Valence	Valence	
2^1^ *T*_2_	7.292	Valence	Valence	4^1^ *A*_1_	8.471	Valence	Valence	
2^1^ *E*	7.664	Valence	Valence	5^1^ *A*_1_+6^1^ *A*_2_	8.947	Valence	Valence	
3^1^ *T*_1_	8.082	Valence	Valence	5^1^ *A*_2_	8.774	Nonvalence	Mixed	
3^1^ *E*	8.239	Valence	Valence	8^1^ *A*_1_+8^1^ *A*_2_	9.426	Valence	Valence	
1^1^ *A*_1_	8.264	Valence	Valence	6^1^ *A*_1_	9.326	Valence	Valence	
4^1^ *T*_1_	8.403	Nonvalence	Rydberg	7^1^ *A*_2_	9.128	Valence	Valence	
3^1^ *T*_2_	8.469	Valence	Valence	7^1^ *A*_1_	9.418	Nonvalence	Mixed	8.92(9)^b^
5^1^ *T*_1_	8.865	Valence	Valence					
4^1^ *T*_2_	9.048	Valence	Valence	9^1^ *A*_1_	9.659	Nonvalence	Mixed	9.343
5^1^ *T*_2_	9.339	Nonvalence	Mixed					9.652
4^1^ *E*	9.366	Nonvalence	Mixed	10^1^ *A*_1_+9^1^ *A*_2_	9.785	Nonvalence	Mixed	
6^1^ *T*_1_	9.479	Nonvalence	Mixed					
2^1^ *A*_2_	9.506	Nonvalence	Mixed					

a The assignments are performed based on the stabilization method (SM) and from the orbital occupation analysis (orbitals). Here, only one component of degenerate states is presented for calculation performed with td-TD-DFT. For EOM-CCSD, only states of *A*_1_ and *A*_2_ symmetry, according to the *C_2v_* point group, are presented. Experimental photoabsorption bands at that are mentioned in the text are also shown.^10^

b The last decimal of the energy values is given in brackets for the less resolved features.

Measurements of the high-resolution photoabsorption spectra of CCl_4_ have recently been reported in the literature.^10^ These spectra display predominantly broad features up to 9.2 eV, while sharper peaks appear at higher energies. It is well established that valence or mixed excited states with a large valence contribution typically give rise to broad structures in photoabsorption spectra, whereas sharp features are usually attributed to excitations to Rydberg or mixed states with a large Rydberg component or to vibrational progressions. Our proposed state assignments agree with the experimental observations: the states below 9.2 eV are predominantly valence in character, while those at higher energies are either mixed valence-Rydberg or Rydberg in nature. Since CCl_4_ belongs to the *T*_*d*_ symmetry group, only transitions to states of *T*_2_ symmetry are dipole-allowed upon photoabsorption. Consequently, the nonvalence states identified below 9.2 eV in our analysis do not contribute to the observed spectrum, and no sharp peak associated with these are seen.

### HCOOH

The stabilization graphs for the electronically excited states of HCOOH, calculated at the TD-DFT/CAM-B3LYP/aug-cc-pVDZ level, are presented in Figure 3. Unlike CCl_4_, HCOOH exhibits low-lying mixed states with strong Rydberg components.^11^ Therefore, the stabilization method proposed here identifies only two valence states: states 1^1^ *A*” and 5^1^ *A*” (according to the numbering of the standard basis set). State 1^1^ *A*” does not cross any other state as α varies and is clearly stable. In contrast, state 5^1^ *A*” undergoes avoided crossings with other states between α = 1.10 and α = 1.50. After these crossings, the structure remains stable as the basis set becomes more compact, as expected for a valence state. The remaining states are either Rydberg or mixed states, where the Rydberg component plays a significant role in their description. It is worth noting that the stabilization curves of different Rydberg and mixed states exhibit distinct slopes as the basis set is made more compact. These slopes reflect the extent to which the Rydberg character influences the character of the electronically excited states. States with a stronger Rydberg component, hence, more diffuse in nature, diverge more rapidly than mixed states with substantial valence character as the basis set contracts. Thus, mixed and Rydberg states may also have avoided crossing among themselves. Note, for instance, states 3^1^ *A*’ and 2^1^ *A*” of HCOOH (Figure 3).

**Figure 3:**
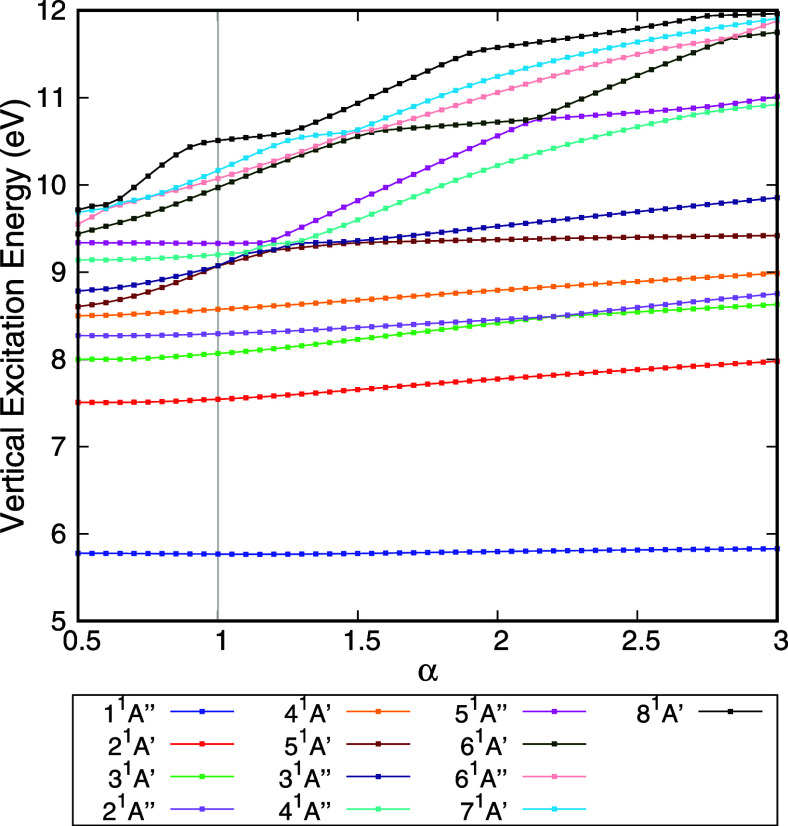
Vertical excitation energy of the electronically excited states of HCOOH as a function of the stabilization parameter (α). States 1 ^1^ *A"* and 5 ^1^ *A"*, following the nomenclature in the default basis set, are valence states. The remaining states are Rydberg or mixed states with a non-negligible Rydberg component. The distinct slopes of the Rydberg and mixed states are associated with the contribution of the Rydberg component to the state. More diffuse states diverge more rapidly than states where the Rydberg component is not as strong as the basis set is compacted.

Since HCOOH is the smallest molecule studied in this work, we also carried out additional calculations using the larger aug-cc-pVTZ basis set and the EOM-CCSD method. The vertical excitation energies obtained with the aug-cc-pVTZ basis set and the CAM-B3LYP functional are shown in the left panel of Figure 4. Due to the more diffuse character of the augmented functions in aug-cc-pVTZ compared to aug-cc-pVDZ, we extended the stabilization calculations up to α = 4 to ensure that the smaller basis set acts as a truly compact one. In general, the upper limit of α should be chosen such that the exponent of the most diffuse function of each angular momentum type in the basis set matches that of the second most diffuse function of the same type. For the aug-cc-pVXZ basis set family, this condition ensures that the most compact basis set becomes comparable in diffuseness to the corresponding cc-pVXZ basis set. Some numerical instabilities may be seen in the stabilization plots (Figure 4), which result in an energy shift of some states for some values of α. These troublesome points do not hamper the ability to perform the assignments. Two valence states are identified, namely states 1^1^ *A*” and 5^1^ *A*”. These results are consistent with those obtained using the smaller aug-cc-pVDZ basis set shown in Figure 3. The stabilization plot for HCOOH obtained with the EOM-CCSD method and the aug-cc-pVDZ basis set is shown in the right panel of Figure 4. Once again, two valence states are identified in the spectrum, states 1^1^ *A*” and 5^1^ *A*”. Thus, the physical conclusions taken using the proposed procedure with larger basis sets and other methods are consistent among themselves.

**Figure 4:**
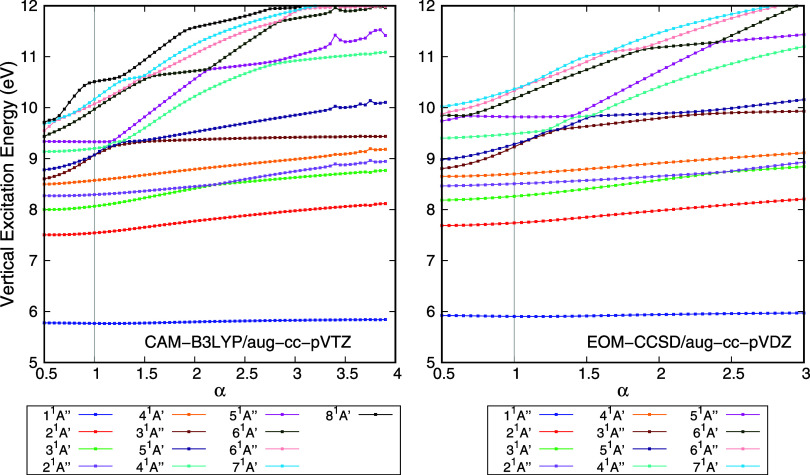
Vertical excitation energy of the electronically excited states of HCOOH as a function of the stabilization parameter (α). States 1^1^ *A*” and 5^1^ *A*”, following the nomenclature in the default basis set, are valence states. Left panel: Calculations were performed with the TD-DFT method, CAM-B3LYP functional, and the aug-cc-pVTZ basis set. Right panel: Calculations were performed with the EOM-CCSD method and the aug-cc-pVTZ basis set.

In Table 2, we compare the assignments made using the present proposal and the assignments performed with a traditional analysis of the character of the orbital to which the electron is excited. Both methods classify the first electronically excited state as a valence state in every calculation performed. However, while the SM identifies a second valence state, the orbital analysis method assigns this state as mixed (state 5^1^ *A*”). According to the TD-DFT calculation performed at the equilibrium geometry with the aug-cc-pVDZ basis set, 92% of this state is described by a configuration of valence character and the remaining 8% involving transitions to orbitals of Rydberg or mixed character. A similar situation occurs with the TD-DFT/aug-cc-pVTZ and EOM-CCSD/aug-cc-pVDZ calculations, where this state is characterized as a mixture of valence and Rydberg configurations. The remaining states are assigned as Rydberg or mixed states by the orbital analysis method, in agreement with the stabilization procedure.

**Table 2:** Vertical Excitation Energy (in eV) and Assignments of the Electronically Excited States of HCOOH^a^

TD-DFT/CAM-B3LYP/aug-cc-pVDZ				TD-DFT/CAM-B3LYP/aug-cc-pVTZ				EOM-CCSD/aug-cc-pVDZ				
State	Energy	SM	Orbitals	State	Energy	SM	Orbitals	State	Energy	SM	Orbitals	Exp.^11^
1^1^ *A*”	5.767	Valence	Valence	1^1^ *A*”	5.784	Valence	Valence	1^1^ *A*”	5.904	Valence	Valence	5.910
2^1^ *A*’	7.542	Nonvalence	Mixed	2^1^ *A*’	7.560	Nonvalence	Mixed	2^1^ *A*’	7.737	Nonvalence	Mixed	
3^1^ *A*’	8.066	Nonvalence	Mixed	3^1^ *A*’	8.074	Nonvalence	Mixed	3^1^ *A*’	8.260	Nonvalence	Mixed	
2^1^ *A*”	8.293	Nonvalence	Rydberg	2^1^ *A*”	8.319	Nonvalence	Rydberg	2^1^ *A*”	8.506	Nonvalence	Rydberg	
4^1^ *A*’	8.573	Nonvalence	Mixed	4^1^ *A*’	8.550	Nonvalence	Mixed	4^1^ *A*’	8.698	Nonvalence	Mixed	
5^1^ *A*’	9.070	Nonvalence	Rydberg	5^1^ *A*’	9.001	Nonvalence	Mixed	5^1^ *A*’	9.282	Nonvalence	Mixed	
3^1^ *A*”	9.072	Nonvalence	Mixed	3^1^ *A*”	8.939	Nonvalence	Rydberg	3^1^ *A*”	9.229	Nonvalence	Mixed	
4^1^ *A*”	9.200	Nonvalence	Mixed	4^1^ *A*”	9.202	Nonvalence	Mixed	4^1^ *A*”	9.488	Nonvalence	Mixed	
5^1^ *A*”	9.330	Valence	Mixed	5^1^ *A*”	9.314	Valence	Mixed	5^1^ *A*”	9.819	Valence	Mixed	8.8 to 9.3
6^1^ *A*’	9.969	Nonvalence	Rydberg	6^1^ *A*’	9.815	Nonvalence	Rydberg	6^1^ *A*’	10.177	Nonvalence	Rydberg	
6^1^ *A*”	10.072	Nonvalence	Mixed	6^1^ *A*”	9.993	Nonvalence	Rydberg	6^1^ *A*”	10.366	Nonvalence	Mixed	
7^1^ *A*’	10.165	Nonvalence	Rydberg	7^1^ *A*’	10.021	Nonvalence	Mixed	7^1^ *A*’	10.331	Nonvalence	Rydberg	
8^1^ *A*’	10.509	Nonvalence	Rydberg	8^1^ *A*’	10.268	Nonvalence	Rydberg					

a The assignments are made through the stabilization method (SM) and from the orbital analysis (orbital). Experimental photoabsorption bands at that are mentioned in the text are also shown.^11^ Note that the second valence state is associated with the broad background in the photoabsorption band.

The second valence state identified through the SM procedure warrants further discussion. This corresponds to state 5^1^ *A*”, obtained using the default basis set at the equilibrium geometry. As is shown in Figure 5 from ref. 11, the vertical excitation energy of the states between 7.5 and 9.5 eV is sensitive to the C=O bond length. The authors performed calculations at the TD-DFT/CAM-B3LYP/aug-cc-pVDZ level.^11^ At the equilibrium geometry, state 5^1^ *A*” is nearly degenerate with state 4^1^ *A*”, which is a Rydberg state. As the bond contracts, the energy difference between state 5^1^ *A*” and the lower Rydberg state increases, and state 5^1^ *A*” evolves into a purely valence state. Conversely, as the bond length increases, state 5^1^ *A*″ undergoes avoided crossings with other low-lying states, evolving into a purely valence character. In other words, the nuclear motion decouples the valence state from its neighboring Rydberg states, which decreases configuration mixing up to a point where a purely valence state is identified. This PEC provides clear evidence that there is a valence state in this energy region. Additional experimental evidence supporting the presence of a valence state in this energy range comes from the broad background observed in the photoabsorption cross section between 8.8 and 9.3 eV.^11^ Due to the proximity of this valence state to neighboring Rydberg states at equilibrium geometry, configurations of a Rydberg character influence the description of this state, and single-point electronic structure calculations incorrectly assigned to it a mixed character. With the SM approach proposed here, the valence character assignment is direct and does not require the computation of potential energy curves or surfaces since the decoupling of the valence and Rydberg states comes from the variation of the stabilization parameter. It is important to highlight that PEC computations require either prior knowledge of the nuclear trajectory or a thorough scan of multiple PECs. In contrast, the only variable in the stabilization procedure proposed here is the stabilization parameter itself, which drives the decoupling of the states. As such, our approach offers a more direct and efficient way to obtain the nature of the excited states in relation to the PECs computations. Thus, as in the case of CCl_4_, the stabilization method once again enables a systematic assignment of the valence electronically excited states of HCOOH, without the need for subjective orbital analysis or PEC computations.

**Figure 5:**
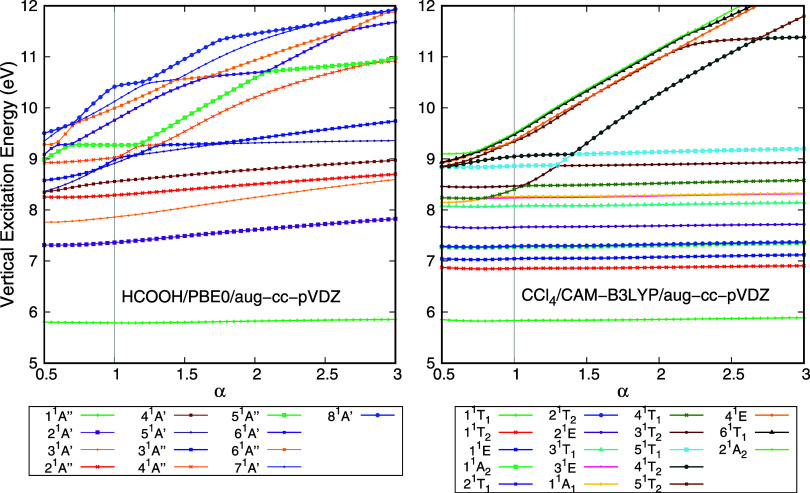
Vertical excitation energy of the electronically excited states of CCl_4_ (left panel) and formic acid (right panel) as a function of the stabilization parameter (α). Calculations were performed with the TD-DFT method, the aug-cc-pVTZ basis set, and CAM-B3LYP for HCOOH and PBE0 for CCl_4_.

As noted in the previous paragraph, the photoabsorption cross section of HCOOH exhibits a broad background feature associated with valence states around 8.8 and 9.3 eV.^11^ The first absorption band, spanning from 4.5 to 6.5 eV, is well separated from the rest of the spectrum and displays a broad profile, which is attributed to the first valence excitation of HCOOH. The photoabsorption bands between 6.5 and 8.8 eV are assigned to mixed states with a high valence component, with some vibrational progressions.^11^ Note that the slopes of the states in the stabilization graphs (Figures 3 and 4) in this energy regime are small compared with the slopes of purely Rydberg states. This is an indication that these states indeed have a strong valence character, with some Rydberg contributions from other configurations. Aside from the background already discussed, the higher-energy region of the experimental spectrum features sharp structures, which are attributed to Rydberg and mixed states as well as vibrational progressions^11^ in agreement with the assignments presented here.

As a final point, because our proposed approach is method-independent, any functional should work and provide at least qualitative analogous results. To verify this, calculations were performed for CCl_4_ and formic acid with the functionals interchanged, that is, using PBE0 for HCOOH and CAM-B3LYP for CCl_4_. These results are listed in Figure 5. As expected, the assignments obtained with these functionals are analogous to the ones presented previously.

### 2-Chlorotoluene

The vertical excitation energies of the 20 lowest electronically excited states of 2-chlorotoluene are shown in Figure 6. These calculations were performed by using the B3LYP functional combined with the aug-cc-pVDZ basis set. Since 2-chlorotoluene is larger than the molecule previously discussed, its spectrum is denser, and as a result, a narrower energy range is presented here compared to the earlier cases. Nevertheless, as with the smaller molecule, it is possible to identify states 2^1^ *A*’, 3^1^ *A*’, 8^1^ *A*”, and 11^1^ *A*” (following the nomenclature with α = 1) in Figure 6 as valence states, given that their energies do not vary significantly with the stabilization parameter α. The remaining states are characterized as either mixed or Rydberg states. These results demonstrate that our method remains effective for larger molecules with dense excitation spectral features.

**Figure 6:**
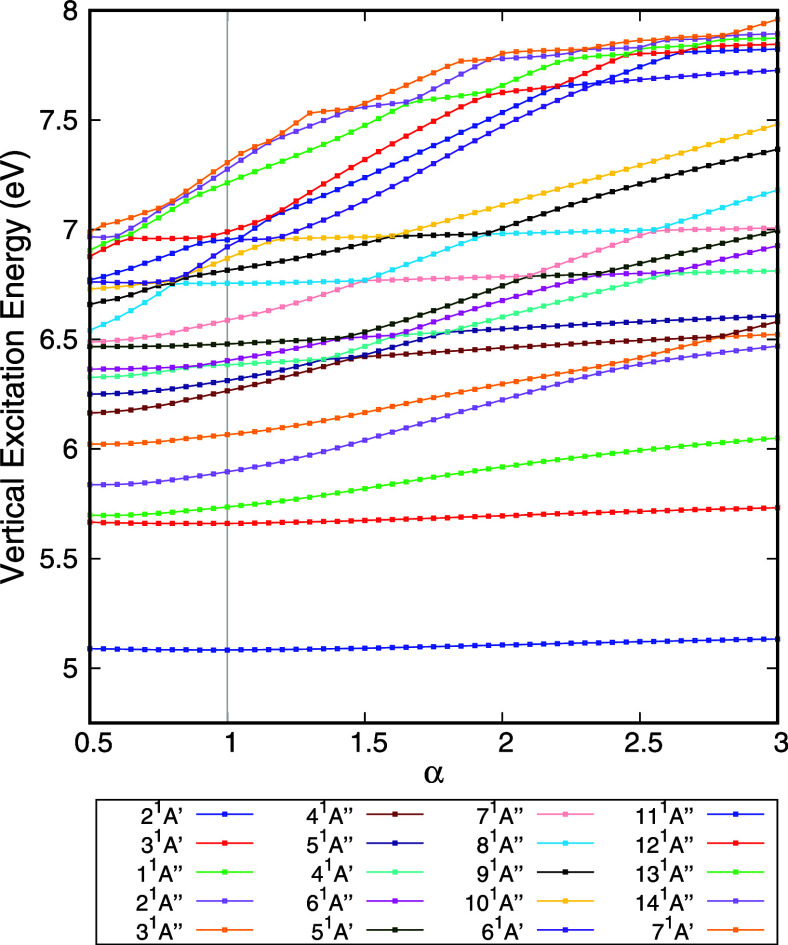
Vertical excitation energy of the electronically excited states of 2-chlorotoluene as a function of the stabilization parameter (α). Calculations were performed with the TD-DFT method, B3LYP functional, and the aug-cc-pVTZ basis set.

In Table 3, a comparison is presented between the assignments obtained by using the proposed method and those based on traditional orbital analysis. As shown, discrepancies are observed only for states 4^1^ *A*’ and 5^1^ *A*’. Although these states are assigned as valence states in the literature,^17^ it is important to note that valence configurations account for at most 88% of their description.^17^ The remaining 12% involve transitions to Rydberg or mixed orbitals. Therefore, while they were previously classified as valence states, they are, in fact, of mixed character with a dominant valence component. This less acurate assignment highlights the inherent subjectivity of the orbital analysis procedure: the threshold of valence contribution required to classify a state as purely valence is not rigorously defined. In contrast, our method readily identifies these states as nonvalence with a strong valence character, based on the stabilization behavior. Specifically, the slopes of these states in the stabilization plot are smaller than those of the higher-lying Rydberg states (Figure 6).

**Table 3:** Vertical Excitation Energy (in eV) and Assignments of the Electronically Excited States of 2-Chlorotoluene^a^

TD-DFT/B3LYP/aug-cc-pVDZ				
State	Energy	SM	Orbitals	Exp.^17^
2^1^ *A*’	5.083	Valence	Valence	4.688
3^1^ *A*’	5.660	Valence	Valence	5.93
1^1^ *A*”	5.735	Nonvalence	Mixed	
2^1^ *A*”	5.896	Nonvalence	Mixed	
3^1^ *A*”	6.065	Nonvalence	Mixed	
4^1^ *A*”	6.265	Nonvalence	Rydberg	
5^1^ *A*”	6.312	Nonvalence	Rydberg	
4^1^ *A*’	6.384	Nonvalence	Valence	6.64(4)^b^
6^1^ *A*”	6.403	Nonvalence	Mixed	
5^1^ *A*’	6.479	Nonvalence	Valence	6.64(4)^b^
7^1^ *A*”	6.587	Nonvalence	Rydberg	
8^1^ *A*”	6.756	Valence	Valence	Bg.
9^1^ *A*”	6.815	Nonvalence	Mixed	
10^1^ *A*”	6.869	Nonvalence	Rydberg	
6^1^ *A*’	6.922	Nonvalence	Mixed	
11^1^ *A*”	6.954	Valence	Valence	Bg.
12^1^ *A*”	6.990	Nonvalence	Rydberg	
13^1^ *A*”	7.214	Nonvalence	Rydberg	
14^1^ *A*”	7.276	Nonvalence	Mixed	
7^1^ *A*’	7.306	Nonvalence	Rydberg	

a The assignments are performed based on the stabilization method (SM) and from the orbital occupation analysis (orbitals). See the text for the discussion regarding the different assignments of states 4^1^ *A*’ and 5^1^ *A’*. Experimental photoabsorption bands at that are mentioned in the text are also shown.^11^ States 8^1^ *A”* and 11^1^ *A”* contribute mainly to the photoabsorption cross section’s background (Bg.).

b The last decimal of the energy values is given in brackets for the less resolved features.

The photoabsorption spectrum of 2-chlorotoluene has recently been reported in the literature.^17^ Between 4.4 and 5.2 eV, a broad band with vibrational progression is observed, associated with the first valence state. In the 5.4 to 6.2 eV range, a broad shoulder, superimposed by small sharp features, appears. The underlying broad structure is attributed to the second valence state, while the sharp features are linked to vibrational progressions and Rydberg or mixed states present in this energy region. The intense feature around 6.6 eV is attributed to transitions to mixed states with a strong valence character (states 4^1^ *A*’ and 5^1^ *A*’). The remaining valence states contribute mainly to the background of the photoabsorption cross section, while the higher-lying nonvalence states are associated with the sharper structures observed at higher photon energies.^17^

## Conclusion

In this work, we propose a novel and systematic approach to distinguish valence electronically excited states from mixed and Rydberg state of molecules using the stabilization method.^14^ Valence states remain stable as the basis set becomes more compact, whereas Rydberg and mixed states with a non-negligible Rydberg component tend to diverge. We applied this method to three test cases: CCl_4_, HCOOH and 2-chlorotoluene. The characterization of electronically excited states obtained through our approach shows good agreement with that of the traditional orbital analysis method. The only discrepancies were observed for HCOOH and 2-chlorotoluene. For HCOOH, the stabilization method identified two valence states, while the orbital analysis procedure made in the equilibrium geometry misinterpreted and predicted only one. For 2-chlorotoluene, the subjectiveness of the orbital analysis led to the wrongful assignment of two mixed states as valence states. In contrast, the stabilization procedure proposed here readily identifies these states as mixed with a strong valence component. With this new systematic approach, we aim to enhance the characterization of electronically excited states by reducing the arbitrariness involved in and with traditional methods.
